# Effect of Stenosis Severity on Outcomes After Rescue Stenting for Acute Middle Cerebral Artery Occlusions: a Real-world Multicenter Analysis

**DOI:** 10.1007/s00062-026-01635-7

**Published:** 2026-02-27

**Authors:** Andrea Maria Alexandre, Luca Scarcia, Valerio Brunetti, Arturo Consoli, Wen Sun, Yingjie Xu, Xianjun Huang, Charlotte Chung, Alessandro Sgreccia, Mohamad Abdalkader, Nicola Limbucci, Francesco Arba, Alessandro Pedicelli, Maria Maddalena Viola, Luigi Cirillo, Mariangela Piano, Vittorio Semeraro, Emilio Lozupone, Chiara Gaudino, Riccardo Russo, Julien Burel, Julien Allard, Nicolas Chausson, Roberta Partesano, Nicola Cavasin, Nicolò Mandruzzato, Joseph Domenico Gabrieli, Pietro Trombatore, Antonio Armando Caragliano, Federico Mazzacane, Giancarlo Salsano, Antioco Sanna, Pietro Panni, Andrea Zini, Frédéric Clarençon, Eytan Raz, Thanh Nguyen, Aldobrando Broccolini

**Affiliations:** 1 Interventional Neuroradiology Unit, Fondazione Policlinico Universitario A. Gemelli IRCCS, Rome, Italy; 2 Department of Neuroradiology, Hôpitaux Universitaires Henri-Mondor, Créteil, France; 3 Neurology Unit, Fondazione Policlinico Universitario A. Gemelli IRCCS, Rome, Italy; 4 Department of Diagnostic and Therapeutic Neuroradiology, Hôpital Foch, Suresnes, France; 5 Department of Neurology, The First Affiliated Hospital of USTC, Hefei, China; 6 Department of Neurology, First Affiliated Hospital of Wannan Medical College, Wuhu, China; 7 Department of Neurosurgery and Department of Radiology, New York University Langone Medical Center, New York, United States; 8 Department of Radiology and Neurology, Boston Medical Center, Boston, United States; 9 Interventional Neurovascular Unit, Azienda Ospedaliero-Universitaria Careggi, Florence, Italy; 10 Stroke Unit, Azienda Ospedaliero-Universitaria Careggi, Florence, Italy; 11 Department of Neurology and Stroke Center, IRCCS Istituto delle Scienze Neurologiche di Bologna, Maggiore Hospital, Bologna, Italy; 12 Neuroradiology Unit, IRCCS Istituto delle Scienze Neurologiche di Bologna, Maggiore Hospital, Bologna, Italy; 13 Neuroradiology Unit, ASST Grande Ospedale Metropolitano Niguarda, Milan, Italy; 14 Interventional Radiology Unit, P.O.C. SS Annunziata, Taranto, Italy; 15 Neuroradiology Unit, Ospedale Vito Fazzi, Lecce, Italy; 16 Department of Neuroradiology, Policlinico Umberto I, Rome, Italy; 17 Neuroradiology Unit, Azienda Ospedaliera Citta’ della Salute e della Scienza di Torino, Turin, Italy; 18 Department of Radiology, Centre Hospitalier Universitaire de Rouen, Rouen, France; 19 Department of Interventional Neuroradiology, Pitié-Salpêtrière Hospital, Paris, France; 20 Department of Neurology, Centre Hospitalier Sud Francilien, Corbeil-Essonnes, France; 21 Neuroradiology Unit, Ospedale di Parma, Parma, Italy; 22 Neuroradiology Unit, Ospedale dell’ Angelo, Mestre, Italy; 23 Interventional Neuroradiology, Azienda Ospedaliera Universitaria Integrata Verona, Verona, Italy; 24 Neuroradiology Unit, Azienda Ospedaliera di Padova, Padova, Italy; 25 Department of Diagnostic Imaging and of Interventional Radiology and Neuroradiology, Ospedale Garibaldi, Catania, Italy; 26 Neuroradiology Unit, Azienda Ospedaliera Universitaria Policlinico “G. Martino”, Messina, Italy; 27 Department of Stroke Unit and Emergency Neurology, Fondazione Istituto Neurologico Nazionale Casimiro Mondino, Pavia, Italy; 28 Neuroradiology Unit, IRCCS Ospedale Policlinico San Martino, Genoa, Italy; 29 Neuroradiology Unit, Ospedale Santa Corona, Pietra Ligure, Italy; 30 Interventional Neuroradiology Unit, IRCCS Ospedale San Raffaele, Milan, Italy; 31 Department of Neuroscience, Catholic University of the Sacred Heart, Rome, Italy

**Keywords:** Acute ischemic stroke, Large vessel occlusion, Intracranial artery stenosis, Rescue stenting, Clinical outcome

## Abstract

**Purpose:**

Rescue stenting (RS) can achieve durable recanalization in cases of acute large vessel occlusion due to underlying intracranial artery stenosis (ICAS), but its clinical effects may be influenced by procedural factors. This study aimed to evaluate whether the severity of stenosis affects the outcomes after RS.

**Methods:**

In this multicenter retrospective study, patients with acute middle cerebral artery occlusion and underlying ICAS were divided into two groups based on the treatment they received: mechanical thrombectomy (MT) + RS (*n* = 172) or MT-only (*n* = 131). Inverse probability of treatment weighting was used to balance baseline characteristics. We systematically evaluated stenosis thresholds from 40% to 90% to identify the optimal cutoff that best differentiated treatment effects on the 90-day modified Rankin Scale (mRS) score and safety outcomes, including symptomatic intracranial hemorrhage (sICH).

**Results:**

A stenosis severity of 75% was identified as the optimal cutoff for effect modification. While RS improved recanalization rates overall, its effect on the 90-day mRS score was beneficial only in patients with > 75% stenosis compared to MT-only (Average Treatment Effect (ATE) −0.98, 95% CI −1.73 to −0.22; *p* = 0.01). In contrast, it showed a detrimental effect in those with < 75% stenosis (ATE 1.08, 95% CI 0.32 to 1.83; *p* = 0.005). Furthermore, RS increased the rate of sICH regardless of ICAS severity.

**Conclusions:**

The clinical benefit of RS is contingent on the underlying stenosis severity, providing favorable outcomes in patients with high-grade stenoses only. ICAS severity should also be considered for treatment decisions, though these findings require validation in prospective controlled studies.

**Supplementary Information:**

The online version of this article (10.1007/s00062-026-01635-7) contains supplementary material, which is available to authorized users.

## Introduction

Mechanical thrombectomy (MT) is the standard of care for anterior circulation large vessel occlusion (LVO), achieving higher recanalization rates than medical therapy [1]. Nonetheless, MT can fail in a subset of patients, often because of underlying intracranial artery stenosis (ICAS). Intracranial artery stenosis accounts for up to 10% of acute LVOs (and an even higher proportion in the Asian population) and is associated with higher rates of procedural failure, potentially affecting long-term outcomes [2–4]. Rescue therapies such as stenting have emerged as pivotal strategies for achieving durable recanalization in this patient population. Evidence from retrospective studies and meta-analyses has shown that rescue stenting (RS) is particularly beneficial in MT-refractory occlusion, whereas patients with residual stenosis after MT have no significant benefit [5–9]. However, a recent trial failed to demonstrate a clear benefit of balloon angioplasty with or without stenting in patients with unsuccessful recanalization or residual stenosis > 70% following MT, compared to those treated with MT alone [10]. Therefore, the decision to perform RS remains challenging owing to the scarcity of randomized trial data and procedure-related risks, including hemorrhage, stent thrombosis, and vessel damage. In real-world practice, neurointerventionalists generally base their decisions on intraprocedural factors such as immediate re-occlusion, impaired distal flow, lesion morphology, and technical feasibility [11–13]. However, information on how stenosis grade affects outcomes is limited, and clarifying this relationship may help to refine patient selection for stenting in routine clinical practice.

In this retrospective, multicenter, real-world study, we investigated whether the severity of intracranial stenosis modifies the effect of RS on the clinical, procedural, and safety outcomes in patients with acute middle cerebral artery (MCA) occlusion due to underlying ICAS.

## Methods

### Patients and Treatment

This retrospective observational study involved 25 comprehensive stroke centers across Europe, the United States, and China and was conducted within the framework of a non-profit study protocol approved by the Ethics Committee of the Coordinating Center. The local ethics committee approved the use of the patient data. The need for informed consent was waived due to the retrospective nature of the study and because all therapeutic procedures were routine care. The analysis was conducted in adherence with the STrengthening the Reporting of OBservational studies in Epidemiology statement [14].

The prospective databases of the participating centers were screened for consecutive patients with acute MCA occlusion who underwent endovascular treatment between January 2020 and June 2024. Mechanical thrombectomy was performed with a stent-retriever, direct contact aspiration, or a combined technique. Only patients with underlying ICAS in the M1 or proximal M2 segment of the MCA identified during MT were included in the study. The decision to perform RS after MT was at the discretion of the neurointerventionalist or based on local protocols. MCA stenosis degree was calculated on post-thrombectomy angiograms using the following formula: (1-(D-stenosis/D-normal)) × 100, where D‑stenosis is the diameter of the artery at the site of the most severe stenosis and D‑normal is the diameter of the proximal normal artery [15, 16]. Radiological and angiographic data were reviewed locally by neuroradiologists/neurointerventionalists blinded to clinical information. Patients were divided into two groups based on whether they received MT only (MT-only group) or MT followed by RS (MT + RS group).

### Measures of Outcome

Clinical outcomes were assessed using the modified Rankin Scale (mRS) acquired at 90 days after stroke. The primary outcome measure was the ordinal 90-day mRS score. Secondary clinical outcome measures were the dichotomized 90-day mRS scores of 0–2 and 0–3. Efficacy and safety outcome measures included: 1) successful recanalization at the end of the endovascular procedure, defined as a modified Treatment In Cerebral Infarction (mTICI) score of 2b–3; 2) post-procedure re-occlusion of the target vessel; 3) occurrence of symptomatic intracranial hemorrhage (sICH, any parenchymal hematoma associated with an increase of ≥ 4 points in the National Institutes of Health Stroke Scale—NIHSS—score or ≥ 2 points in an NIHSS subcategory, according to the Heidelberg bleeding classification) [17]; and 4) 90-day mortality.

### Statistical Analysis

The baseline characteristics were summarized using descriptive statistics. Continuous variables were reported as medians and interquartile ranges (IQR), and categorical variables as counts and percentages. Between-group differences were assessed using Fisher’s exact test for categorical variables and Mann-Whitney U test for continuous variables.

We used inverse probability of treatment weighting (IPTW) with overlap weights to balance baseline characteristics between the treatment groups. The model incorporated covariates selected a priori for clinical relevance (age, sex, admission NIHSS score, baseline ASPECTS, baseline mRS, occlusion site (M1 vs. M2 segment), stenosis grade) plus additional imbalanced variables identified in preliminary analyses. Importantly, recanalization status after initial MT (mTICI 2b–3 vs. 0–2a) was also included. This covariate corresponded to the final recanalization status for the MT-only group and the immediate pre-stenting status for the MT + RS group, allowing isolation of the effect of stenting on outcomes, whether performed for persistent occlusion (mTICI 0–2a) or for a flow-limiting residual stenosis after successful recanalization (mTICI 2b–3) [6]. Covariate balance was assessed using absolute standardized mean differences (SMD), with an SMD < 0.1 indicating adequate balance.

The primary effect measure was the average treatment effect (ATE) of MT + RS vs. MT-only on the ordinal 90-day mRS score, estimated using a weighted linear model. For dichotomous outcomes, the effect was estimated as relative risk (RR), representing the ratio of outcome probabilities between treatment groups, with the MT-only group serving as the reference. All models incorporated overlap weights and employed robust variance estimators.

Two complementary approaches were used to examine whether stenosis severity modified the treatment effect of RS. First, to identify a clinically operational cutoff in the absence of prior data, we systematically tested stenosis thresholds from 40% to 90% in 5% increments using IPTW regression models with the 90-day mRS score as the outcome. The optimal stenosis cutoff was defined empirically as the value yielding the largest absolute difference in treatment effects between patients with stenosis above versus below that threshold. Second, a confirmatory analysis was performed treating stenosis as a continuous variable. An IPTW-adjusted linear model was fitted, including an interaction term between treatment and continuous stenosis percentage. A significant interaction (*p* < 0.05) would indicate effect modification across the stenosis spectrum. From this model, the stenosis threshold where the treatment effect transitioned from harmful to beneficial was estimated. For all outcome measures, effect modification was assessed by including an interaction term (stenting × dichotomized stenosis severity) in the final regression models. Subgroup-specific treatment effects and their 95% confidence intervals were derived using linear combinations of coefficients, with the *p*-value for the interaction term serving as the test for effect modification. For comparison, unadjusted (unweighted) analyses were performed for all the outcomes. Finally, a sensitivity analysis was performed to account for potential center effects using a mixed-effects model with the center included as a random intercept. This approach assessed whether the primary results were sensitive to unmeasured center-level factors that could influence the outcomes.

Statistical significance was set at *p* < 0.05. All analyses were performed using R software v.4.3.2, with the *lmtest, sandwich, cobalt*, and *CBPS* packages (https://www.r-project.org).

## Results

A total of 18,732 patients were screened, of whom 541 underwent endovascular treatment for acute MCA ICAS-LVO. After excluding those treated with rescue strategies other than stenting and cases with missing data on IPTW variables, 303 patients were included in the final analysis: 172 treated with MT + RS and 131 with MT only. A flow diagram of patient enrollment is shown in Supplementary Fig. 1. Univariate analysis of baseline, procedural, and outcome characteristics between the two treatment groups is reported in Table 1. Compared with the MT-only group, the MT + RS group had higher rates of hypertension, diabetes, and general anesthesia use, higher median ASPECTS, more severe MCA stenosis, and lower median NIHSS score, whereas atrial fibrillation was more common in the MT-only group. As expected with bailout stenting, the rates of successful recanalization after the initial MT were lower in patients receiving MT + RS. Diverse peri-procedural antiplatelet protocols were used in patients receiving RS, most commonly involving a glycoprotein IIb/IIIa inhibitor (tirofiban or abciximab), administered either alone (45.3% of patients) or in combination with other antiplatelet agents (20.2% of patients). Inverse probability weighting was adjusted for imbalanced variables in univariate analysis, along with the other prespecified covariates (Supplementary Table 1).

**Table 1:** Univariate analysis of demographic, baseline characteristics, procedural features and outcomes

	MT-only (*N* = 131)	MT + RS (*N* = 172)	p*
**Demographic and baseline characteristics**			
Age, median (IQR)	71 (63–78)	71 (61–77)	0.41
Sex (female), n/*N* (%)	65/131 (49.6)	70/172 (40.7)	0.12
Hypertension, n/*N* (%)	78/131 (59.5)	125/172 (72.7)	**0.02**
Dyslipidemia, n/*N* (%)	49/131 (37.4)	68/172 (39.5)	0.71
Diabetes, n/*N* (%)	24/131 (18.3)	51/172 (29.7)	**0.02**
Atrial fibrillation, n/*N* (%)	50/131 (38.2)	29/172 (16.9)	**<** **0.001**
Coronary artery disease, n/*N* (%)	18/131 (13.7)	31/172 (18.0)	0.31
Prior stroke, n/*N* (%)	23/131 (17.5)	30/172 (17.4)	0.98
Prior antiplatelets, n/*N* (%)	22/128 (21.9)	43/172 (25.0)	0.10
Prior anticoagulants, n/*N* (%)	16/129 (12.4)	25/172 (14.5)	0.60
Pre-event mRS score, median (IQR)	0 (0–0)	0 (0–0)	0.59
NIHSS score, median (IQR)	14 (9–18)	12 (7–17)	**0.04**
Left-side stroke, n/N (%)	73/131 (55.7)	93/172 (54.1)	0.77
M1 segment occlusion, n/N (%)	111/131 (84.7)	147/172 (85.5)	0.86
ASPECTS, median (IQR)	8 (7–9)	9 (8–10)	**<** **0.001**
**Procedural data**			
IVT, n/N (%)	30/131 (22.9)	45/172 (26.2)	0.52
General anesthesia, n/N (%)	58/131 (44.3)	106/172 (61.6)	**0.003**
Onset-to-end of procedure (minutes), median (IQR)	450 (320–600)	400 (270–600)	0.12
Degree of MCA stenosis (%), median (IQR)	40 (30–80)	80 (70–90)	**<** **0.001**
mTICI 2b–3 at the end of MT, n/N (%)	105/131 (80.2)	64/172 (37.2)	**<** **0.001**
Type of peri-procedural antiplatelet agents, n/N (%)			
None	–	8/172 (4.7)	–
Aspirin	–	17/172 (9.8)	–
Cangrelor	–	12/172 (6.9)	–
GPI	–	78/172 (45.3)	–
Clopidogrel	–	4/172 (2.3)	–
Aspirin + clopidogrel	–	12/172 (6.9)	–
Aspirin + GPI	–	24/172 (13.9)	–
Aspirin + clopidogrel + GPI	–	9/172 (5.2)	–
Clopidogrel + GPI	–	2/172 (1.1)	–
Aspirin + cangrelor	–	6/172 (3.4)	–
**90-day clinical outcomes**			
mRS score, median (IQR)	3 (1–5)	3 (1–4)	0.65
mRS score 0–2, n/*N* (%)	64/131 (48.8)	84/172 (48.8)	0.99
mRS score 0–3, n/*N* (%)	77/131 (58.8)	112/172 (65.1)	0.26
**Efficacy and safety outcomes**			
Final mTICI 2b–3, n/*N* (%)	105/131 (80.2)	164/172 (95.3)	**<** **0.001**
Post-procedure re-occlusion, n/*N* (%)	2/87 (2.3)	15/149 (10.1)	**0.03**
sICH, n/*N* (%)	6/131 (4.6)	20/172 (11.6)	**0.03**
90-day mortality, n/*N* (%)	20/131 (15.3)	21/172 (12.2)	0.45

*ICAS* intracranial artery stenosis; *MT* mechanical thrombectomy; *RS* rescue stenting; *mRS* modified Rankin scale; *IQR* interquartile range; *GPI* Glycoprotein IIb/IIIa inhibitor; *NIHSS* National Institutes of Health Stroke Scale; *ASPECTS* Alberta Stroke Program Early CT score; *IVT* intravenous thrombolysis; *MCA* middle cerebral artery; *mTICI* modified Treatment in Cerebral Infarction; *PH* parenchymal hemorrhage; *sICH* symptomatic intracranial hemorrhage

* significance set at *p* < 0.05

Evaluation of stenosis cutoffs from 40% to 90% showed significant effect modification of RS vs. MT-only by stenosis severity, with a 75% cutoff demonstrating the largest difference in treatment effect between patients with stenosis above versus below this threshold (difference of ATEs = −2.06, *p*-interaction < 0.001; Supplementary Table 2). The confirmatory continuous analysis aligned with this threshold, showing a significant linear interaction between RS and stenosis severity (interaction β = −0.028 per 1% stenosis increase, 95% CI −0.047 to −0.009; *p* = 0.004), with the ATE crossing from harmful to beneficial at 70.3% stenosis (Supplementary Fig. 2). The empirically determined 75% cutoff value was used in all subsequent analyses for clinical interpretability and consistency with the exploratory findings.

**Fig. 1:**
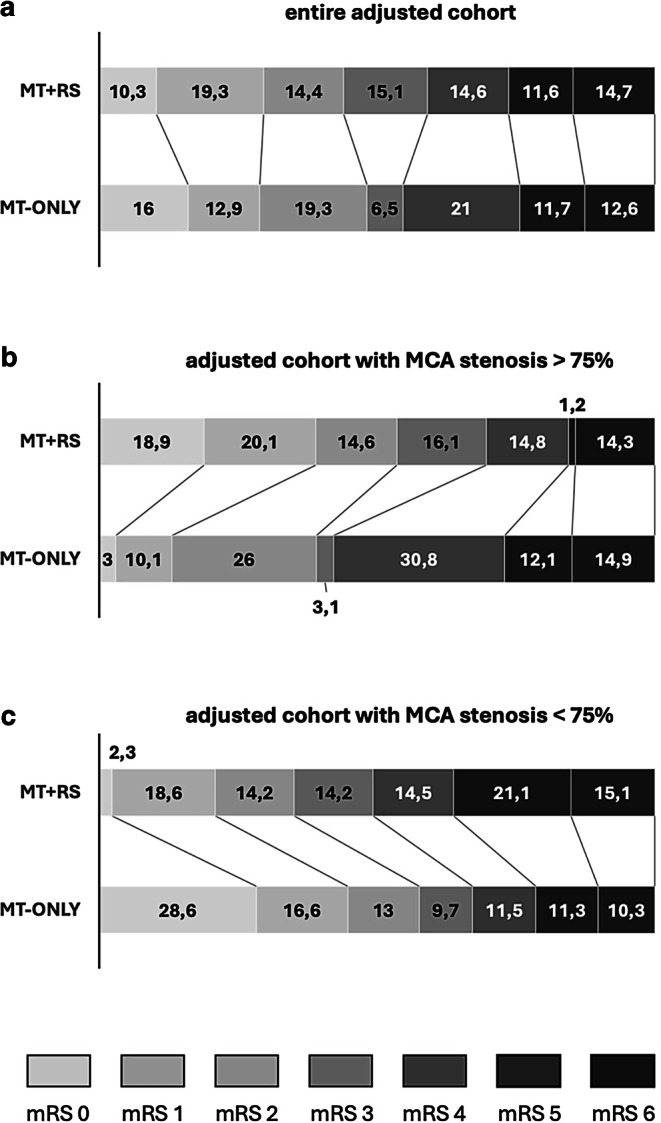
Distribution of 90-day modified Rankin Scale scores by treatment group and stenosis severity. **a** Modified Rankin Scale (mRS) score distribution in the entire cohort after inverse probability of treatment weighting (IPTW). **b**–**c** IPTW-adjusted mRS distributions stratified by the underlying severity of the intracranial stenosis, demonstrating the differential treatment effect of rescue stenting in (**b**) patients with stenosis > 75% and (**c**) patients with stenosis < 75%. Values represent percentages of patients at each mRS score

Despite a significant increase in successful recanalization in the overall population (adjusted RR 1.65, 95% CI 1.37–1.98; *p* < 0.001), which was more pronounced in the > 75% stenosis subgroup (*p*-interaction < 0.001), clinical outcomes diverged by stenosis severity. Compared to MT-only, stenting was associated with improved outcomes in patients with > 75% stenosis (adjusted ATE −0.98, 95% CI −1.73 to −0.22; *p* = 0.01) but with worse outcomes in patients with < 75% stenosis (adjusted ATE 1.08, 95% CI 0.32 to 1.83; *p* = 0.005). Figure 1 shows the 90-day mRS scores distribution according to treatment group and stenosis severity.

Dichotomized clinical outcomes showed similar effect modification by stenosis severity (*p*-interaction < 0.001 for both mRS 0–2 and 0–3). For mRS 0–2, stenting was associated with higher point estimates for favorable outcome in patients with > 75% stenosis, although this difference did not reach statistical significance (adjusted RR 1.79, 95% CI 0.77–4.17; *p* = 0.17), and with a significantly lower likelihood of favorable outcome in patients with < 75% stenosis (adjusted RR 0.40, 95% CI 0.19–0.88; *p* = 0.02). For mRS 0–3, stenting benefited > 75% stenosis (adjusted RR 3.09, 95% CI 1.31–7.29; *p* = 0.01) and showed marginally worse outcomes in patients with < 75% stenosis (adjusted RR 0.48, 95% CI 0.22–1.06; *p* = 0.07).

For safety outcomes, stenting was associated with an increased risk of sICH in the overall cohort (adjusted RR 5.15, 95% CI 1.78–14.86; *p* = 0.002), without evidence of effect modification by stenosis severity (*p*-interaction = 0.90). The risk increase was statistically significant in patients with < 75% stenosis (adjusted RR 5.0, 95% CI 1.54–16.28; *p* = 0.01) and was of similar magnitude but did not reach statistical significance in patients with > 75% stenosis (adjusted RR 5.33, 95% CI 0.66–42.95; *p* = 0.11). No differences in the rates of post-procedural re-occlusion and 90-day mortality were observed overall or across the subgroups. The results of all analyses are reported in Table 2.

**Table 2:** Effect of rescue stenting on outcomes

	Unadjusted					IPTW-adjusted				
	ATE	RR	95% CI	*p*	*p*-int*	ATE	RR	95% CI	*p*	*p*-int*
*90-day mRS score*										
Entire cohort	−0.12	–	−0.58–0.33	0.60	–	0.09	–	−0.35–0.54	0.68	**–**
< 75% stenosis	0.68	–	0.04–1.32	**0.04**	**<** **0.001**	1.08	–	0.32–1.83	**0.005**	**<** **0.001**
> 75% stenosis	−1.03	–	−1.71–−0.35	**0.004**		−0.98	–	−1.73–−0.22	**0.01**	
*90-day mRS 0–2*										
Entire cohort	–	1.00	0.63–1.58	0.99	–	–	0.84	0.48–1.47	0.54	**–**
< 75% stenosis	–	0.65	0.28–1.07	0.08	**0.02**	–	0.40	0.19–0.88	**0.02**	**0.01**
> 75% stenosis	–	1.83	0.84–4.10	0.13		–	1.79	0.77–4.17	0.17	
*90-day mRS 0–3*										
Entire cohort	–	1.31	0.82–2.09	0.26	–	–	1.18	0.66–2.11	0.57	**–**
< 75% stenosis	–	0.64	0.33–1.25	0.19	**0.002**	–	0.48	0.22–1.06	0.07	**0.002**
> 75% stenosis	–	3.27	1.48–7.39	**0.004**		–	3.09	1.31–7.29	**0.01**	
*mTICI 2b–3*										
Entire cohort	–	1.19	0.93–1.52	0.16	–	–	1.65	1.37–1.98	**<** **0.001**	**–**
< 75% stenosis	–	1.04	0.74–1.45	0.82	**0.02**	–	1.17	1.03–1.33	**0.01**	**<** **0.001**
> 75% stenosis	–	2.24	1.33–4.08	**0.004**		–	2.86	1.78–4.60	**<** **0.001**	
*Post-procedure re-occlusion*										
Entire cohort	–	4.38	1.24–27.79	**0.04**	–	–	1.63	0.22–12.12	0.63	**–**
< 75% stenosis	–	6.96	1.12–133.43	0.07	0.19	–	7.71	0.69–85.86	0.10	0.25
> 75% stenosis	–	0.97	0.19–17.76	0.97		–	1.07	0.10–11.26	0.95	
*sICH*										
Entire cohort	–	2.54	1.08–6.94	**0.04**	–	–	5.15	1.78–14.86	**0.002**	**–**
< 75% stenosis	–	3.50	1.24–11.24	**0.02**	0.86	–	5.00	1.54–16.28	**0.01**	0.90
> 75% stenosis	–	2.84	0.54–52.21	0.31		–	5.33	0.66–42.95	0.11	
*90-day mortality*										
Entire cohort	–	0.78	0.40–1.51	0.45	–	–	1.22	0.52–2.85	0.65	**–**
< 75% stenosis	–	0.94	0.36–2.34	0.90	0.70	–	1.57	0.51–4.83	0.43	0.56
> 75% stenosis	–	0.71	0.24–2.35	0.54		–	0.97	0.29–3.25	0.95	

*IPTW* inverse probability of treatment weighting; *ATE* average treatment effect; *RR* relative risk; *CI* confidence interval; p‑int. *p*-interaction; *mRS* modified Rankin Scale; *mTICI* modified Treatment In Cerebral Infarction; *sICH* symptomatic intracerebral hemorrhage

* significance set at *p* < 0.05

Sensitivity analysis adjusted for center effects supported these findings, with stenting showing benefits in the > 75% stenosis subgroup and detrimental effects in the < 75% stenosis subgroup for the primary outcome. For both dichotomized outcome measures, stenting demonstrated more pronounced benefits in the high-grade stenosis subgroup than in the primary analysis (Supplementary Table 3). For sICH occurrence and re-occlusion, precise RRs estimation remained limited by the reduced sample size and few events.

## Discussion

Our multicenter retrospective analysis demonstrated that the effect of RS vs. MT alone on clinical outcomes was modified by the severity of the underlying MCA stenosis.

From a technical standpoint, RS has emerged as an effective adjunct to MT for ICAS-LVO, stabilizing stenosis and improving angiographic results [5, 9, 18, 19]. However, its clinical impact remains less certain, as the treatment effect can be modified by procedural factors. Rescue stenting appears to be most beneficial in MT-refractory occlusion [5–8], whereas it offers no clear advantage when residual stenosis persists after effective MT [6]. To separate the effect of stenting itself from that of the initial angiographic result, our analysis incorporated the immediate post-MT recanalization status as a covariate. This approach showed that stenosis severity is an important effect modifier, and systematic cutoff analysis identified 75% as the optimal threshold. Accordingly, stenting was associated with an improved mRS score particularly in patients with > 75% stenosis. In contrast, in patients with less severe stenosis, stenting was linked to worse functional outcomes, despite higher rates of angiographic success. These findings indicate that the decision to pursue RS should be guided not only by the immediate angiographic result of MT but also by the underlying severity of stenosis. The observation that benefits emerge primarily in severe vessel disease aligns with data from non-acute symptomatic ICAS patients, where stenting becomes potentially superior to medical management only when stenosis exceeds approximately 85% [20]. The discrepant impact of RS across stenosis severities can be partly explained by differences in the hemodynamic advantage conferred by stenting in conjunction with procedural hazards, such as sICH. Indeed, in our cohort, stenting was associated with an overall increased risk of sICH without evidence of effect modification by stenosis severity. In patients with more severe stenosis, stenting likely provides a meaningful gain in luminal caliber, which translates into improved mRS scores despite hemorrhagic complications. In contrast, in patients with moderate stenosis a limited hemodynamic improvement may be outweighed by bleeding risk. However, the hemorrhagic safety profile of RS remains unclear. Although some studies have reported improved outcomes without increased sICH, the ANGEL-REBOOT trial found higher hemorrhagic complications following rescue therapies [5, 7, 10]. This variability may reflect context-dependent risks influenced by angiographic scenario, stenosis severity, and antiplatelet regimens. Specifically, stenting after failed MT appears to improve reperfusion without elevating hemorrhage risk, whereas stenting for residual stenosis after successful thrombectomy is associated with higher sICH rates [6]. Collectively, these findings underscore the need for standardized protocols to better define safety profiles across different clinical contexts.

Additional mechanisms consistent with futile reperfusion may contribute to poorer outcomes in patients with less severe stenosis, in whom the benefits of recanalization after RS may be offset by microvascular injury or distal embolization [21, 22]. In these patients, vessel instability and a tendency toward re-occlusion due to residual thrombus may be more relevant than fixed stenosis, limiting the hemodynamic benefit of stent deployment while exposing patients to risks such as perforator compromise or endothelial injury [23]. Data on predictors of futile recanalization after acute rescue therapy for ICAS-LVO remain limited [24, 25], and the role of stenosis severity has not been examined. Our findings underscore the limitations of angiographic recanalization as a surrogate for clinical success and suggest that stenosis severity may help identify patients in whom the risks of stenting outweigh its benefits, highlighting the need for more refined patient selection beyond recanalization status alone.

Finally, stenting in moderately narrowed vessels could paradoxically increase the post-procedure re-occlusion risk compared to MT alone, whereas in severe stenosis the baseline re-occlusion risk is already elevated and not necessarily worsened by stenting. However, we did not find a significant association between RS and the occurrence of post-procedural re-occlusion overall or across subgroups in the IPTW-adjusted analysis. This result requires cautious interpretation due to missing data for this outcome and the inability to account for post-procedural antiplatelet therapies.

The primary limitation of our study is its retrospective design, which introduces the potential for unmeasured confounders. Despite IPTW, residual confounding, particularly by indication, is likely because the decision to perform RS was based on operator judgment and nuanced angiographic factors not captured in our registry data, such as vessel instability or specific lesion characteristics (e.g., calcification, plaque eccentricity or morphology). The absence of a centralized core laboratory for angiographic review also introduces the potential for measurement variability. In addition, stenosis severity was assessed after thrombectomy once antegrade flow had been restored; because vessel caliber may change following clot retrieval, this may have influenced stenosis measurements and introduced additional variability.

Second, the 75% stenosis cutoff was empirically derived from our dataset. While this threshold closely aligns with the crossing point estimated by our confirmatory continuous analysis and is biologically plausible, this data-driven approach must be considered hypothesis-generating, and the specific threshold requires external validation.

Third, heterogeneity in endovascular techniques, devices and peri-procedural management across the 25 centers represents an additional source of bias. In particular, the wide variability in antiplatelet regimens (including single agents, dual antiplatelets, and glycoprotein IIb/IIIa inhibitors alone or in combination) limited the possibility to explore their potential effects on outcomes in our analysis. Finally, while we adjusted for recanalization status after initial MT to isolate the effect of stenting, this adjustment may not fully account for differences in procedural complexity between groups. However, our study design reflects real-world practice and reports on the outcomes of RS procedures that were performed, possibly including contexts where supporting evidence remains limited. This provides valuable data on the potential risks of stenting in suboptimal candidates.

## Conclusions

Our findings demonstrate that the benefit of RS after MT is influenced by the severity of underlying stenosis. This evidence supports individualized decision-making, with particular caution in moderate stenosis, where stenting benefits must be carefully weighed against procedural risks. Randomized trials are needed to provide definitive guidance and to clarify the most appropriate use of this treatment approach.

## Supplementary Information

ESM1: Supplementary material 1

## Data Availability

No datasets were generated or analysed during the current study.
