# Supplementary material for: Effect of Stenosis Severity on Outcomes After Rescue Stenting for Acute Middle Cerebral Artery Occlusions: a Real-world Multicenter Analysis
Source: Clin Neuroradiol. 2026 Feb 27;36(2):711–20. doi: 10.1007/s00062-026-01635-7 (PMC13319141; doi:10.1007/s00062-026-01635-7)
Supplement: Supplementary file 1 — ESM1: Supplementary material 1 [file 62_2026_1635_MOESM1_ESM.pdf]

Effect of stenosis severity on outcomes after rescue stenting for acute middle cerebral artery occlusions: a real-world multicenter analysis

Supplementary Material

Supplementary Figure 1. Flow diagram of patient enrollment

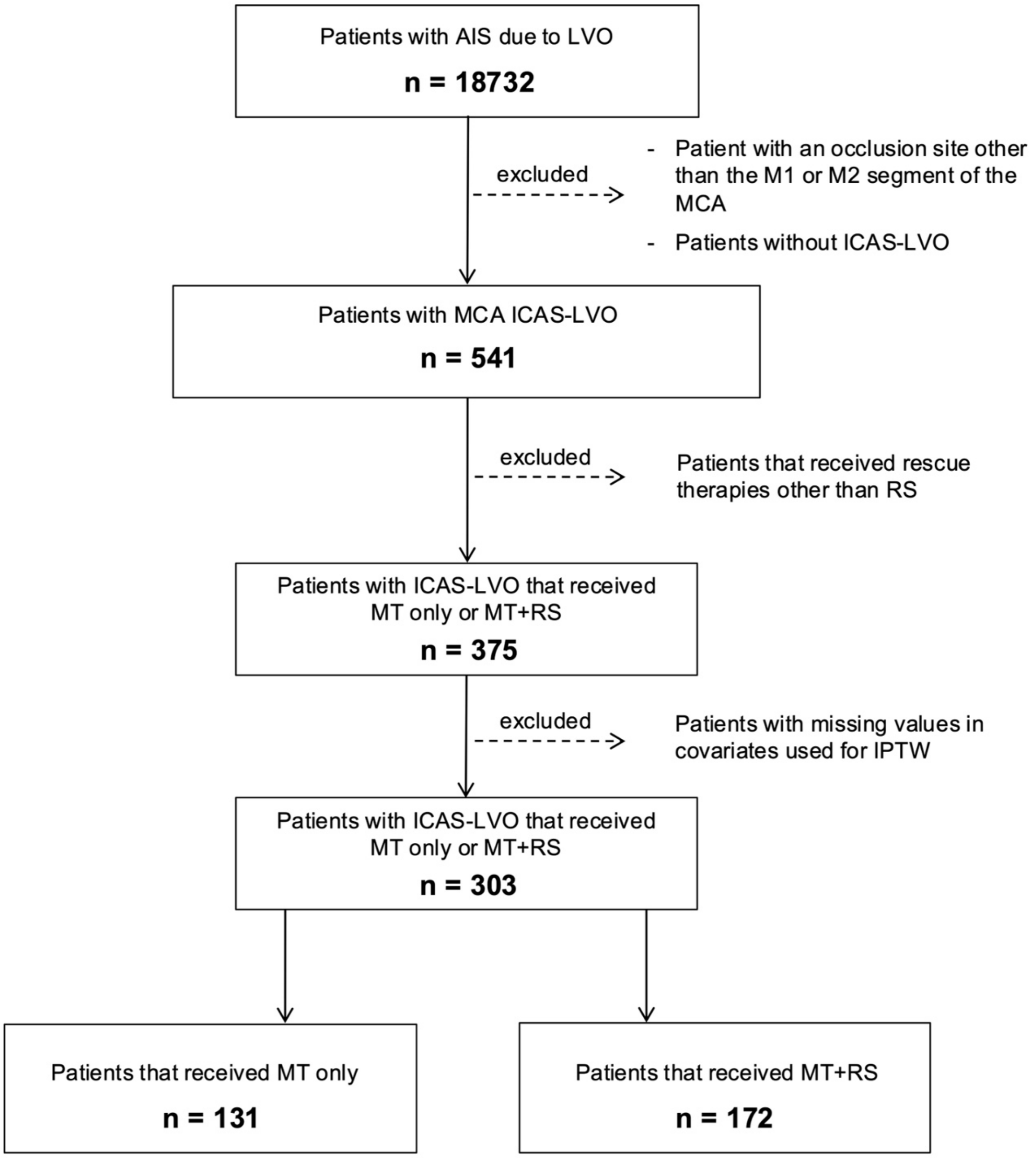

AIS, acute ischemic stroke; LVO, large vessel occlusion; MCA, middle cerebral artery; ICAS, intracranial artery stenosis; RS, rescue stenting; MT, mechanical thrombectomy; IPTW, inverse probability of treatment weighting.

Supplementary Table 1. Covariate balance before and after inverse probability of treatment weighting

	Standardized mean difference	
	before IPTW	after IPTW
age	-0.096	<0.001
sex	-0.180	<0.001
pre-event mRS score	0.063	<0.001
baseline NIHSS score	-0.229	<0.001
baseline ASPECTS	0.489	<0.001
M1 occlusion	-0.021	<0.001
hypertension	0.280	<0.001
diabetes	0.268	<0.001
atrial fibrillation	-0.491	<0.001
degree of MCA stenosis	1.114	<0.001
general anesthesia	0.353	<0.001
mTICI 2b-3 score at the end of MT	-0.969	<0.001

IPTW, inverse probability of treatment weighting; mRS, modified Rankin Scale; NIHSS, National Institutes of Health Stroke Scale; ASPECTS, Alberta Stroke program early CT score; MCA, middle cerebral artery; mTICI, modified Thrombolysis in Cerebral Infarction; MT, mechanical thrombectomy.

Supplementary Table 2. Effect of rescue stenting versus mechanical thrombectomy on the 90-day mRS score across different stenosis grades

	ATE	95% CI	p*	ATE difference	p-int*
<40% stenosis	+1.02	-0.08 - 2.12	0.07	1.29	0.05
>40% stenosis	-0.26	-0.92 - 0.40	0.43		
<45% stenosis	+1.02	-0.08 - 2.12	0.07	1.29	0.05
>45% stenosis	-0.26	-0.92 - 0.40	0.43		
<50% stenosis	+1.11	0.17 - 2.05	**0.02**	1.53	**0.01**
>50% stenosis	-0.42	-1.11 - 0.28	0.24		
<55% stenosis	+1.14	0.21 - 2.07	**0.02**	1.57	**0.01**
>55% stenosis	-0.43	-1.13 - 0.27	0.23		
<60% stenosis	+1.02	0.21 - 1.84	**0.01**	1.53	**0.006**
>60% stenosis	-0.50	-1.23 - 0.22	0.17		
<65% stenosis	+1.00	0.18 - 1.82	**0.03**	1.51	**0.007**
>65% stenosis	-0.51	-1.25 - 0.22	0.17		
<70% stenosis	+1.10	0.30 - 1.90	**0.08**	1.90	**<0.001**
>70% stenosis	-0.81	-1.54 - -0.07	**0.03**		
<75% stenosis	+1.08	0.32 - 1.83	**0.005**	2.05	**<0.001**
>75% stenosis	-0.98	-1.73 - -0.22	**0.01**		
<80% stenosis	+0.60	-0.09 - 1.28	0.09	1.45	**0.01**
>80% stenosis	-0.85	-1.74 - 0.03	0.06		
<85% stenosis	+0.56	-0.11 - 1.24	0.10	1.41	**0.01**
>85% stenosis	-0.84	-1.76 - 0.08	0.07		
<90% stenosis	+0.43	-0.18 - 1.04	0.16	1.77	**0.004**
>90% stenosis	-1.33	-2.04 - -0.27	**0.02**		

ATE, average treatment effect; CI, confidence interval; p-int., p-interaction; * Significance set at p<0.05.

Supplementary Figure 2. Treatment effect modification by stenosis severity

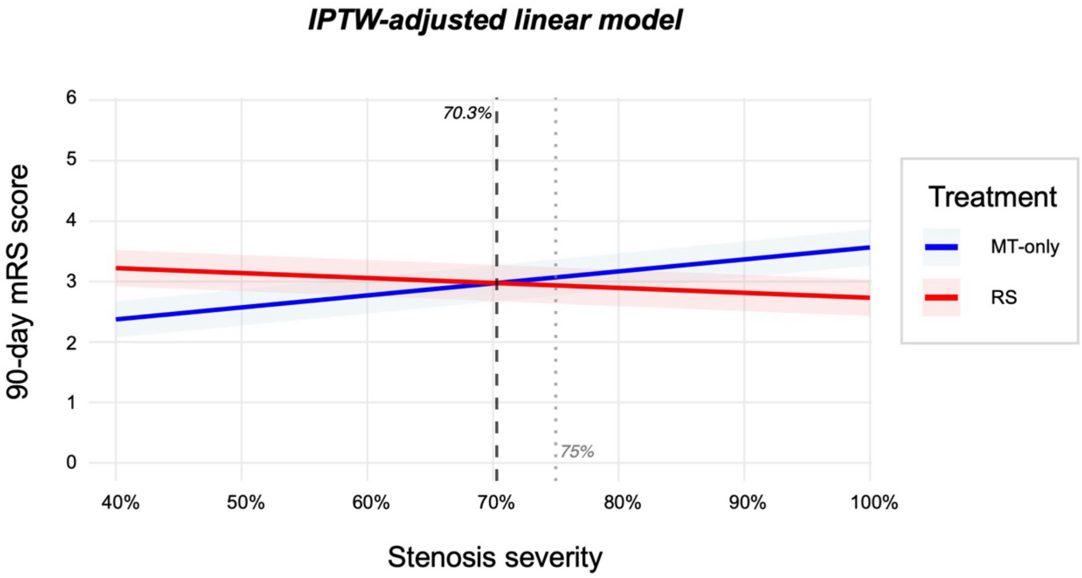

	Estimate (β) #	95% CI	p *
Treatment × stenosis interaction	-0.028	-0.047 to -0.009	0.004

Continuous analysis confirmed a significant linear interaction, with the ATE crossing from harmful to beneficial at 70.3% stenosis.

IPTW, inversion probability of treatment weighting; mRS, modified Rankin Scale; MT, mechanical thrombectomy; RS, rescue stenting; CI, confidence interval; #, estimate per 1% stenosis increase; *, significance set at $p<0.05$.

Supplementary Table 3. Effect of stenting on outcomes: sensitivity analysis with center random effect

	IPTW-adjusted				
**outcome measure**	**ATE**	**RR**	**95% CI**	**p ***	**p-int.***
**90-day mRS**					
Entire cohort	-0.03		-0.41 - 0.35	0.86	
<75% stenosis	0.88		0.36 - 1.41	**0.001**	**<0.001**
>75% stenosis	-0.82		-1.35 - -0.29	**0.003**	
**90-day mRS 0-2**					
Entire cohort		1.20	0.94 - 1.43	0.13	
<75% stenosis		0.60	0.30 - 1.01	0.06	**<0.001**
>75% stenosis		1.72	1.30 - 2.05	**0.001**	
**90-day mRS 0-3**					
Entire cohort		1.35	1.18 - 1.68	**0.003**	
<75% stenosis		0.88	0.52 - 1.20	0.50	**<0.001**
>75% stenosis		1.98	1.61 - 2.18	**<0.001**	
**Final mTICI 2b-3**					
Entire cohort		1.46	1.13 - 1.89	**0.004**	
<75% stenosis		1.10	0.78 - 1.54	0.60	**0.02**
>75% stenosis		2.07	1.37 - 3.12	**<0.001**	
**Post-procedure re-occlusion**					
Entire cohort		1.25	0.22 - 6.52	0.79	
<75% stenosis		-	-	-	-
>75% stenosis		-	-	-	
**sICH**					
Entire cohort		9.65	2.64 - 17.91	**0.002**	
<75% stenosis		7.81	1.91 - 15.72	**0.006**	-
>75% stenosis		-	-	-	
**90-day mRS 6**					
Entire cohort		1.79	0.54 - 1.97	0.82	
<75% stenosis		3.08	0.91 - 5.43	0.07	**0.02**
>75% stenosis		0.38	0.09 - 1.36	0.15	

IPW, inverse probability of treatment weighting; ATE, average treatment effect; RR, risk ratio; CI, confidence interval; p-int., p-for-interaction; mRS, modified Rankin Scale; mTICI, modified Treatment In Cerebral Infarction; sICH, symptomatic intracranial hemorrhage; *, significance set at $p < 0.05$.

For post-procedure re-occlusion and sICH, precise RRs estimation remained limited by reduced sample size and few events.
